# Investigation of Infantile Scurvy

**Published:** 1897-09

**Authors:** 


### INVESTIGATION OF INFANTILE SCURVY.

The American Pediatric Society is making a Collective Investigation of Infantile Scurvy as occurring in North America, and earnestly requests the co-operation of physicians through their sending of reports of cases, whether these have already been published or not. No case will be used in such a way as to interfere with its subsequent publication by the observer. Blanks containing questions to be filled out will be furnished on application to any one of the committee. A final printed report of the investigation will be sent to those furnishing cases.

J. P. Crozer Griffith, M. D., *Chairman*, 123 S. 18th St., Philadelphia.

William D. Booker, M. D., 853 Park Ave., Baltimore.

Charles G. Jennings, M. D., 457 Jefferson Ave., Detroit.

Augustus Caille, M. D., 753 Madison Ave., New York City.

J. Lovett Morse, M. D., 317 Marlboro St., Boston.

*Committee.*

